# Fathers’ engagement in pregnancy and childbirth: evidence from a national survey

**DOI:** 10.1186/1471-2393-13-70

**Published:** 2013-03-20

**Authors:** Maggie Redshaw, Jane Henderson

**Affiliations:** 1 Policy Research Unit for Maternal Health and Care, National Perinatal Epidemiology Unit, University of Oxford, Old Road Campus, Headington, Oxford OX3 7LF, UK

**Keywords:** Fathers, Pregnancy, Childbirth, Paternal engagement

## Abstract

**Background:**

Early involvement of fathers with their children has increased in recent times and this is associated with improved cognitive and socio-emotional development of children. Research in the area of father’s engagement with pregnancy and childbirth has mainly focused on white middle-class men and has been mostly qualitative in design. Thus, the aim of this study was to understand who was engaged during pregnancy and childbirth, in what way, and how paternal engagement may influence a woman’s uptake of services, her perceptions of care, and maternal outcomes.

**Methods:**

This study involved secondary analysis of data on 4616 women collected in a 2010 national maternity survey of England asking about their experiences of maternity care, health and well-being up to three months after childbirth, and their partners’ engagement in pregnancy, labour and postnatally. Data were analysed using descriptive statistics, chi-square, binary logistic regression and generalised linear modelling.

**Results:**

Over 80% of fathers were ‘pleased or ‘overjoyed’ in response to their partner’s pregnancy, over half were present for the pregnancy test, for one or more antenatal checks, and almost all were present for ultrasound examinations and for labour. Three-quarters of fathers took paternity leave and, during the postnatal period, most fathers helped with infant care. Paternal engagement was highest in partners of primiparous white women, those living in less deprived areas, and in those whose pregnancy was planned. Greater paternal engagement was positively associated with first contact with health professionals before 12 weeks gestation, having a dating scan, number of antenatal checks, offer and attendance at antenatal classes, and breastfeeding. Paternity leave was also strongly associated with maternal well-being at three months postpartum.

**Conclusions:**

This study demonstrates the considerable sociodemographic variation in partner support and engagement. It is important that health professionals recognise that women in some sociodemographic groups may be less supported by their partner and more reliant on staff and that this may have implications for how women access care.

## Background

Early involvement of fathers with their children has increased in recent times [1]. Such involvement is associated with improved cognitive and socio-emotional development of children [2,3]. Absent fathers have been associated with poorer educational, behavioural and developmental outcomes in children with both direct effects on infant and child behaviour and indirect effects possibly due to partner relationship problems, lack of social support and exposure to increased levels of maternal stress hormones whilst *in utero*[2,4]. Partner support during pregnancy may also encourage healthier maternal behaviour, for example with regard to cigarette smoking and alcohol consumption [2]. Research has indicated that men who feel unready for fatherhood tend to be less involved, find the transition to parenthood more challenging, and may be less likely to be committed fathers [5].

Research in the area of father’s engagement with pregnancy and childbirth has mainly focused on white middle-class men and has been mostly qualitative in design [5]. Such research as exists relating to non-white men and those from lower socioeconomic groups suggests that they experience lower satisfaction with the information provided and with their level of inclusion in decision-making [6]. Research from the USA on African-American families found higher rates of absent fathers than in other ethnic groups, particularly in geographical areas with high unemployment and low income [2]. Contrary to stereotype, after adjusting for employment, it was found that black men were more likely to be involved in housework and childcare than other groups [2] and in this group, the quality of the parenting was directly related to pregnancy intentions [2].

Two UK studies from the 1990s examined the association between socioeconomic status and degree of partner support. They found that ‘working class’ men were less likely to take time off, accompany their partner to clinic appointments, be present in labour, or to help postnatally [7]; and conversely, ‘middle-class’ couples tended to be better prepared for the transition to parenthood, better informed and supported than working class couples, and reported better maternal and child outcomes [8].

Partners may be particularly encouraged to be present for some aspects of antenatal care such as ultrasound scans and other screening tests. In one study, they reported feeling that they were there as supporters, rather than direct participants and if a decision was necessary regarding termination of pregnancy for fetal abnormality, men reported reacting objectively and cognitively, without allowing themselves to become emotionally involved [9]. In another study of parents’ experience of termination for fetal abnormality, more men than women considered that they had received adequate support from their partner but men perceived their friends and family as less supportive and, after hospital discharge, men experienced worse emotional well-being than women [10].

According to a study in the UK in 2000, about one third of men attended antenatal classes with their partners but found them of doubtful value, sometimes leading to unrealistic expectations [6,11,12]. However, when there were specific antenatal groups focussing on men’s needs there were benefits in terms of reduced distress, increased ability to cope and improved relationship with their partner. In a study based in two urban hospitals in the UK it was reported that although married fathers were more likely to attend antenatal classes, those who did attend differed little from those who did not with respect to their experience of childbirth, their emotional wellbeing postnatally or in attachment to their infant [13]. Fathers’ less positive experiences of childbirth were associated with higher depressive symptomatology at six weeks after the birth; however, relationships with pre-existing mental health could not be explored. Using a behavioural style measure, a sub-group of fathers who attended antenatal classes, but who showed a high propensity to avoid ‘threat-relevant’ information reported a more negative experience than a similar group not attending classes.

It seems that almost all women in industrialised countries have their partner with them during labour and birth [6,14]. However, there is still doubt in some quarters about the appropriateness of this [15] and it is argued that the partner’s feelings of anxiety and tension, especially if he is reluctant to be there, may make the labour more difficult. In addition, a small retrospective survey from 1988 which investigated men’s experiences as labour coaches suggested that they found it very stressful and the demands exceeded their capabilities [16]. However, the evidence suggests that women place a high value on their partner’s presence and support in labour, leading to reduced anxiety, less perceived pain, greater satisfaction with the birth experience, lower rates of postnatal depression and improved outcomes in the child [6].

If the couple received continuous professional support, the man was more likely to take an active role, feeling empowered rather than helpless, leading to increased satisfaction and greater involvement with early childcare [5,12]. Psychological support was valued by women in the form of emotional expressions of caring, empathy and sympathy [5]. Some men expressed fears of seeing their partner in pain, of not coping, fainting, panicking, failing to respond appropriately, and of being excluded from decision-making and being useless, especially if this was the first time they had been involved in childbirth or they were relatively young. Some also feared that their partner would have a prolonged or complicated labour, that she or the child would die or that the child would be born handicapped [5,6]. Reporting afterwards, it appeared that men found that the actual experience of supporting their partner in childbirth was better than they expected; they were less distressed and frightened, and more excited and elated [6]. However, if a caesarean section was required, fathers were less likely to have been present and, using an adjective checklist [17], were likely to describe their infants more negatively [13].

Genesoni and Tallandini [18] noted in their literature review on fathers and the transition to parenthood that, in a wide range of contexts, men can experience a tension between needing to be the breadwinner and also wanting to be involved in childcare, though this is also true for working women [1,19]. Involvement in care of the baby during the postnatal period may be unaffected by parity but associated with level of education, social class and income of the father [20]. In the changing context of couple relationships and the work situation for men and women there is little recent quantitative information about the extent of fathers’ levels of involvement in pregnancy, childbirth and postnatally and how this varies with sociodemographic characteristics. Thus, among the population of recent fathers in England, the aim was to understand who was engaged during pregnancy and after birth, how much were they engaged and in what way, and how paternal engagement may influence a woman’s uptake of services, her perceptions of care, and maternal outcomes, especially those associated with health and wellbeing.

## Methods

This study utilised data collected in a 2010 survey of new mothers in England. A random sample of 10,000 women aged 16 years and over who had their baby in a two week period in England in 2009 were selected by the Office for National Statistics from birth registration. Mothers whose babies had died were excluded from the sample. Women were sent a questionnaire, invitation letter, information leaflet and an information sheet in a range of languages in early 2010 when the babies were approximately 3 months of age. Using a tailored reminder system non-respondents were sent a reminder letter after two weeks, a further questionnaire after four weeks, and a last reminder letter four weeks later [14].

The survey collected data on care in the antenatal, intrapartum and postnatal periods and how women perceived their care, as well as on sociodemographic factors, including age, parity, partnership status, age on leaving full-time education, and an area based measure of deprivation (Index of Multiple Deprivation (IMD)) [21]. Limited data were available on partners’ age and country of birth based on birth registration. Details were collected from each mother on partner involvement in the pregnancy, labour and postnatally, along with information about paternity leave and partner views of staff communication at each stage. Validated checklists were used to collect data on worries and concerns about labour and birth [22] and women’s perceptions of care at this time [23]. Women who were not living with their husband or the baby’s father at the time of the survey and same sex parents were excluded from the analyses. The study involved secondary data analysis. The original survey evaluating maternity services in England was passed by the Trent Multi-Centre Research Ethics Committee.

### Paternal engagement scores

Two scores were constructed to estimate extent of father engagement. Firstly, a score was constructed to indicate the degree of father engagement prior to birth based on father’s presence or absence at each of the following: pregnancy test or when pregnancy confirmed; one or more antenatal checks; one or more ultrasound scans; one or more antenatal education classes; and during labour. This score also included paternal involvement in finding information about pregnancy; participation in decision-making about antenatal screening; making a birth plan; finding information about labour and birth; and participation in decision-making during labour. Thus, the overall paternal antenatal and labour engagement score could range from 0–10. Secondly, a score was constructed to reflect fathers’ postnatal engagement based on whether he was involved ‘a great deal’ (score 3), ‘a bit’ (score 2), ‘rarely’(score 1) or ‘not at all’ (score 0) in six activities that included changing nappies, playing with the baby and helping when the baby cries. The postnatal engagement score could range from 0–18. Because it was positively skewed, especially in primiparous women, the value of the postnatal engagement score was squared to normalise the distribution.

### Statistical analysis

Univariate analyses were carried out using Chi-square statistics; binary logistic regression was used to estimate the combined effects of sociodemographic variables on fathers’ reactions to pregnancy, presence at antenatal checks and labour, and involvement in postnatal infant care. Generalised linear modelling was used to determine whether certain pre-defined sociodemographic and care variables were associated with the paternal engagement scores. Significance was set at *p < 0.01* in the univariate analyses due to the size of the dataset and the number of tabulations; thereafter it was set at *p < 0.05*. Analyses were carried out using SPSSX versions 17 and 19.

## Results

A total of 5333 women returned usable questionnaires, a response rate of 55.1%. They reported on 4616 fathers (86%) and their involvement during pregnancy, labour and postnatally. Characteristics of women and their partners are shown in Table 1. Three-quarters of fathers (76%) were born in the UK, six percent in the rest of Europe, and eighteen percent were from other countries. The majority of men were in their 30’s (57%), a quarter in their 20’s (26%), and 16% in their 40’s; only one percent of fathers were teenagers. Maternal age and paternal age were highly correlated.

**Table 1:** Characteristics of women and their partners

	**n**	**%**
*Parity*		
Primiparous	2610	50.1
Multiparous	2603	49.9
*Mother’s age*		
<25	795	15.7
25-34	2953	58.2
35+	1326	26.1
*Father’s age*		
<25	217	9.6
25-34	1327	58.6
35+	721	31.8
*Mother’s Ethnic group*		
White	4405	86.0
Mixed	94	1.8
Asian or Asian British	375	7.3
Black or Black British	185	3.6
Chinese or Other	63	1.2
*Index of Multiple Deprivation (quintiles)*		
1 (least deprived)	1040	20.1
2	1024	19.8
3	1104	21.4
4	966	18.7
5	1036	20.0
*Age woman left full-time education*		
</= 16 years	1176	23.0
>16 years	3948	77.0

A comparison of respondent and non-respondent women showed that respondents were slightly more likely to be older, to be married, to be living in the least deprived areas, to be white and to be born in the United Kingdom [14]. Nevertheless of those responding, 14% were from minority ethnic groups.

### Father’s involvement in pregnancy, childbirth and early parenting

The extent of father involvement overall and by parity is shown in Table 2. Most fathers’ initial reaction to the pregnancy was a positive one, with more than 80% being ‘pleased or ‘overjoyed’; there was no difference between the partners of women who had previously given birth and those for whom this was their first baby. Over half of fathers were present for the pregnancy test or when the pregnancy was confirmed (62%) and for one or more antenatal checks (63%), and almost all (89%) were present for one or more ultrasound examinations and for labour (90%). Mothers having their first baby were more likely to have had their partner present when the pregnancy was confirmed, for antenatal checks, for scans, to attend antenatal classes and be present during labour. They were also more likely to have a partner who accessed information about pregnancy and birth and shared in decision-making in pregnancy and during labour.

**Table 2:** Fathers’ involvement in individual aspects of pregnancy, childbirth and early parenting by parity (excluding same sex and single parents) (%)

	**Primiparous women**	**Multiparous women**	**All women**
	**(n = 2286)**	**(n = 2330)**	**(n = 4616)**
*Pregnancy and labour*			
Overjoyed/pleased in reaction to pregnancy (4199)	82.4	82.6	82.5
Present for pregnancy test* (3121)	66.7	56.6	61.7
Present for 1 or more AN check* (3175)	73.3	51.5	62.5
Present for 1 or more ultrasound scan* (4588)	91.2	86.3	88.8
Present for 1 or more AN class* (1707)	59.9	7.7	34.2
Present for labour* (4657)	91.7	88.3	90.0
Not present for any of the above (125)	2.6	2.5	2.6
*Information and decision-making*			
Involved in finding information about pregnancy* (2044)	54.5	27.0	40.9
Involved in finding information about labour/birth* (1947)	51.8	25.8	38.9
Involved in making a birth plan* (2300)	65.1	50.2	57.7
Involved in decision-making regarding AN screening* (2916)	55.9	35.7	45.8
Involved in decision-making in labour* (2769)	60.0	49.5	54.8
Not involved in any of the above* (815)	11.5	21.7	16.6
*Staff Communication*			
Antenatally staff communication rated as good or very good* (3948)	82.6	78.7	80.7
Intrapartum staff communication rated as good or very good (4326)	87.6	88.0	87.8
Postnatally staff communication rated as good or very good (3653)	73.6	75.6	74.6
*Paternity leave* *			
No time off (808)	24.2	30.7	27.5
Less than two weeks off (744)	13.2	17.3	15.3
Two weeks off (2142)	48.7	40.2	44.4
More than two weeks off (625)	13.9	11.8	12.8
*Father involvement postnatally (a great deal/a bit)*	89.5	84.3	86.9
Nappy changing* (4419)			
Helping/supporting feeding* (4440)	90.1	84.5	87.3
Helping when the baby cries (4731)	93.2	92.9	93.0
Bathing the baby* (3932)	85.3	69.3	77.3
Playing with the baby (4909)	96.9	96.2	96.5
Looking after the baby when mother out/at work (4012)	81.5	76.3	78.9

* p < 0.01 AN antenatal, IP intra-partum, PN postnatal.

Women reported that most fathers felt midwives and doctors communicated well with them during pregnancy (81%), more so during labour (88%) and slightly fewer (75%) after the birth, with little difference by parity.

While three-quarters of fathers took paternity leave (72%), some did not take leave or were unable to do so. Partners of first time mothers were slightly more likely to have taken paternity leave and were more likely to have done so for longer.

During the postnatal period, most fathers helped with infant care, with more than three-quarters changing nappies, bathing, helping or supporting feeding, helping when the baby cried, playing with the baby and looking after the baby when the mother was out or at work. Fathers tended to help more with first babies, especially with nappy changing, bathing and feeding. The most common activity reported for all fathers during the postnatal period was playing with the baby (96%). There was no difference in fathers’ activities by gender of the baby.

### Paternal involvement by sociodemographic characteristics

Some differences in key aspects of father involvement by maternal sociodemographic variables presented by parity are shown in Table 3. Partners of primiparous women aged less than 25 years were significantly less enthusiastic in reaction to the pregnancy (76% overjoyed or pleased compared to 90% in the partners of older women); however, partners of multiparous younger women were more likely to attend antenatal checks.

**Table 3:** Maternal sociodemographic variables and fathers’ involvement in key aspects of pregnancy, birth and childcare by parity, maternal and paternal age (years), black and minority ethnic group status and IMD (Index of Multiple Deprivation) by quintile

	**Father overjoyed or pleased in reaction to pregnancy (%)**	**Father present for 1 or more AN checks (%)**	**Father present for labour (%)**	**Father involved in accessing information and decision-making AN or IP (%)**	**Father involved ‘a great deal’ in all aspects of baby care (%)**
Primiparous:					
Mother’s age					
<25	76.2*	81.9	96.1	88.4	38.6
25-34	88.4	78.0	97.1	92.1	40.7
35+	89.8	74.5	95.1	90.8	42.3
Multiparous:					
Mother’s age					
<25	78.5	64.5*	92.9	74.7	32.3
25-34	85.8	56.2	92.1	76.5	29.1
35+	85.7	48.9	90.0	79.6	26.8
Primiparous:					
Father’s age					
<25	68.7*	82.4	94.9	87.2*	36.2
25-34	88.5	78.1	97.1	93.2	41.5
35+	88.1	76.6	96.1	93.1	40.4
Multiparous:					
Father’s age					
<25	82.9	71.0*	95.7	77.6	32.9
25-34	85.4	56.1	91.6	81.4	30.6
35+	85.6	51.5	90.7	80.8	26.7
Primiparous:					
IMD					
1	89.0*	78.2	97.5	93.0	38.2
2	90.6	77.9	97.6	89.7	43.0
3	83.4	78.3	96.7	92.1	43.0
4	84.3	77.0	96.9	89.3	39.6
5	85.3	79.3	93.9	90.8	38.3
Multiparous:					
IMD					
1	85.7	48.2*	94.1*	79.9	25.5
2	88.9	50.3	93.0	80.8	31.4
3	84.8	59.6	92.4	74.3	29.4
4	83.0	54.6	88.5	76.6	29.2
5	83.1	59.6	87.0	74.4	27.3
Primiparous:					
White	86.2	78.4	97.2*	91.1	42.1*
BME	88.3	75.8	92.6	92.0	31.0
Multiparous:					
White	85.6	53.5	93.1*	77.7	29.7*
BME	84.5	58.2	81.2	76.2	22.4

* p < 0.01 AN Antenatal, IP Intrapartum, IMD(1-least deprived), BME Black and Minority Ethnic group.

Fathers whose partners were primiparous women in the least deprived quintiles on the IMD were significantly more likely to be ‘overjoyed’ or ‘pleased’ in reaction to the pregnancy (89% compared to 85% in the most deprived quintile), and partners of multiparous women in the least deprived quintiles were more likely to be present for labour. However, partners’ attendance at antenatal checks was more likely to occur in deprived areas for multiparous women.

Partners of women of Black or Minority Ethnic (BME) origin were significantly less likely to be present for labour than partners of white women (81% compared to 93% in multiparous white women). In particular, partners of women from Black or Black British backgrounds were significantly less likely to be present.

The different sociodemographic variables are closely linked so binary logistic regression was used to estimate the combined effects on partner’s reaction to pregnancy, presence at antenatal checks and labour, involvement in obtaining information and in decision-making, and in infant care. This showed that maternal age, IMD and parity were all strongly associated with the variables of interest (Table 4). Partners of multiparous women were significantly more likely to have a negative reaction to the pregnancy, be less involved in the pregnancy, were less likely to be present during the labour and involved in infant care postnatally. Partners of women living in deprived areas were more likely to have a negative reaction to the pregnancy but more likely to attend antenatal checks, less likely to be involved in obtaining information or decision-making or be present for labour, but more likely to be involved in infant care. Partners of younger women were less likely to have a negative reaction to the pregnancy, more likely to attend antenatal checks, obtain information and be present for labour. Ethnicity was only associated with presence in labour, partners of BME women being less than half as likely to be present compared to partners of white women (Odds ratio 0.38, 95% confidence interval 0.28, 0.50).

**Table 4:** Sociodemographic factors associated with father involvement in pregnancy and labour: binary logistic regression

**Factors associated with father having a negative reaction to pregnancy**	
*Parity*	*Odds ratio (95% CI)*
Primiparous	reference group
Multiparous	1.23 (1.03, 1.46)*
*Index of multiple deprivation (Quintile, 1- least deprived)*	
1	reference group*
2	0.79 (0.60, 1.05)
3	1.25 (0.97, 1.60)
4	1.27 (0.97, 1.66)
5	1.14 (0.86, 1.50)
*Woman’s age*	
<25 years	reference group
25-34 years	0.49 (0.39, 0.61)*
35+ years	0.48 (0.37, 0.63)*
**Factors associated with father being present for 1 or more antenatal checks**	
*Parity*	*Odds ratio (95% CI)*
Primiparous	reference group
Multiparous	0.35 (0.31, 0.41)*
*Index of multiple deprivation (Quintile, 1- least deprived)*	
1	reference group
2	1.04 (0.86, 1.26)
3	1.28 (1.05, 1.56)*
4	1.08 (0.88, 1.33)
5	1.27 (1.03, 1.58)*
*Woman’s age*	
<25 years	reference group
25-34 years	0.79 (0.63, 0.98)*
35+ years	0.62 (0.49, 0.79)*
**Factors associated with father being present for labour**	
*Parity*	*Odds ratio (95% CI)*
Primiparous	reference group
Multiparous	0.40 (0.30, 0.53)*
*Ethnicity*	
White	reference group
Black and Minority Ethnic	0.38 (0.28, 0.50)*
*Index of multiple deprivation (Quintile, 1- least deprived)*	
1	reference group*
2	0.87 (0.57, 1.34)
3	0.79 (0.52, 1.20)
4	0.64 (0.42, 0.97)*
5	0.52 (0.34, 0.78)*
*Woman’s age*	
<25 years	reference group*
25-34 years	1.05 (0.68, 1.63)
35+ years	0.66 (0.41, 1.04)
**Factors associated with father not being at all involved in obtaining information and decision-making**	
*Parity*	*Odds ratio (95% CI)*
Primiparous	reference group
Multiparous	3.11 (2.54, 3.80)*
*Index of multiple deprivation (Quintile, 1- least deprived)*	
1	reference group
2	1.17 (0.87, 1.56)
3	1.44 (1.09, 1.91)*
4	1.36 (1.01, 1.83)*
5	1.44 (1.07, 1.95)*
*Woman’s age*	
<25 years	reference group
25-34 years	0.76 (0.57, 1.01)
35+ years	0.69 (0.50, 0.94)*
**Factors associated with father being involved in infant care**	
*Parity*	*Odds ratio (95% CI)*
Primiparous	reference group
Multiparous	0.79 (0.68, 0.92)*
*Index of multiple deprivation (Quintile, 1- least deprived)*	
1	reference group
2	1.11 (0.87, 1.41)
3	1.14 (0.90, 1.45)
4	1.35 (1.06, 1.73)*
5	1.56 (1.22, 2.00)*

* p < 0.05 Adjusted for maternal age, ethnicity, parity and Index of Multiple Deprivation.

### Paternal engagement score

The results of the generalised linear modelling are shown in Table 5. Paternal engagement scores were significantly higher in partners of primiparous than multiparous women, and in primiparous women who had received more education or were aged 25–34 years, and men born in the UK. Engagement also tended to be higher in partners of white women and those living in less deprived areas but these differences were not statistically significant. Irrespective of parity, where women reported that it was a planned happy pregnancy, the fathers tended to be significantly more engaged (mean partner engagement score in primiparous women with a planned happy pregnancy 7.24 (95% confidence interval 7.13, 7.35) compared to unplanned unhappy pregnancy 6.19 (95% confidence interval 5.86, 6.53).

**Table 5:** Mean partner engagement score (95% confidence interval) by a) socio-demographic factors and parity, and other factors during b) antenatal, c) intrapartum and d) postnatal periods

	**Primiparous women**		**Multiparous women**	
	**Mean (95% CI)**	Adjusted*p	**Mean (95% CI)**	Adjusted*p
**a) Sociodemographic factors**				
*Mother’s ethnicity*				
White	7.13 (7.03, 7.24)		4.93 (4.83, 5.02)	
*BME*	6.83 (6.50, 7.16)	0.93	4.83 (4.51, 5.14)	0.74
*Index of multiple deprivation (quintile)*				
1	7.30 (7.09, 7.52)		4.92 (4.74, 5.10)	
2	7.12 (6.90, 7.33)		4.96 (4.76, 5.16)	
3	7.18 (6.97, 7.39)		4.99 (4.77, 5.20)	
4	7.03 (6.80, 7.26)		4.88 (4.64, 5.12)	
5	6.82 (6.57, 7.08)	0.16	4.78 (4.54, 5.02)	0.42
*Mother left full time education aged*				
<16 yrs	6.77 (6.52, 7.03)		4.93 (4.79, 5.13)	
*>/= 16 yrs*	7.17 (7.06, 7.28)	0.01	4.91 (4.80, 5.01)	0.92
*Age group (mother’s)*				
<25 yrs	6.72 (6.48, 6.96)		5.33 (4.92, 5.73)	
25-34 yrs	7.21 (7.09, 7.33)		4.94 (4.81, 5.06)	
35+ yrs	7.09 (6.86, 7.33)	0.00	4.79 (4.64, 4.94)	0.01
*Father’s country of birth*				
UK	7.24 (7.13, 7.35)		4.94 (4.83, 5.04)	
Europe	6.01 (5.60, 6.42)		5.02 (4.52, 5.53)	
Rest of the world	6.78 (6.45, 7.11)	0.00	4.75 (4.45, 5.05)	0.48
*Pregnancy planning*				
Planned and happy pregnancy	7.24 (7.13, 7.35)		4.97 (4.87, 5.08)	
Unplanned but happy	6.77 (6.44, 7.11)		4.68 (4.34, 5.02)	
Unplanned and unhappy	6.19 (5.86, 6.53)	0.00	4.60 (4.30, 4.89)	0.02
**b) Antenatal care factors**				
*Booking appointment*				
Before 10 weeks	7.23 (7.11, 7.35)		5.06 (4.93, 5.19)	
Booking at/after 10 weeks	6.89 (6.70, 7.08)	0.33	4.69 (4.54, 4.84)	0.00
*Gestation when healthcare professional 1*^*st*^*seen*				
<12 weeks	7.17 (7.07, 7.28)		4.95 (4.85, 5.05)	
12+ weeks	6.22 (5.79, 6.65)	0.01	4.58 (4.27, 4.89)	0.01
*Number of antenatal checks*				
0-5	6.60 (6.31, 6.89)		4.57 (4.36, 4.78)	
6-8	7.14 (6.97, 7.31)		4.76 (4.60, 4.92)	
9-10	7.07 (6.86, 7.27)		5.10 (4.88, 5.32)	
11 or more	7.29 (7.12, 7.45)	0.05	5.22 (5.04, 5.41)	0.00
Dating scan done	7.13 (7.03, 7.23)		4.95 (4.84, 5.05)	
No dating scan	6.73 (6.37, 7.09)	0.05	4.62 (4.32, 4.92)	0.02
*Antenatal classes*				
Offered	7.20 (7.10, 7.30)		5.03 (4.89, 5.16)	
Not offered	6.27 (5.96, 6.58)	0.01	4.79 (4.66, 4.92)	0.02
Attended	7.48 (7.36, 7.60)		5.72 (5.39, 6.06)	
Not attended	6.53 (6.36, 6.70)	0.00	4.85 (4.72, 4.96)	0.00
*Number of antenatal health problems*				
0,1	7.02 (6.86, 7.18)		4.75 (4.59, 4.91)	
2,3	7.21 (7.05, 7.36)		4.84 (4.69, 4.98)	
4+	7.01 (6.79, 7.22	0.18	5.14 (4.96, 5.32)	0.01
*Worries about labour*				
low	7.21 (6.93, 7.48)		4.70 (4.54, 4.87)	
low-medium	7.08 (6.86, 7.30)		5.00 (4.83, 5.18)	
medium-high	7.24 (7.07, 7.41)		4.90 (4.70, 5.09)	
high	6.92 (6.74, 7.09)	0.16	5.07 (4.84, 5.30)	0.02
*Overall satisfaction with antenatal care*				
Satisfied or very satisfied	7.14 (7.04, 7.24)		4.96 (4.86, 5.06)	
Neither satisfied nor dissatisfied	6.82 (6.41, 7.25)		4.50 (4.18, 4.82)	
Dissatisfied or very dissatisfied	6.70 (6.25, 7.13)	0.16	4.66 (4.22, 5.11)	0.01
**c) Intrapartum factors**				
*Type of delivery*				
Spontaneous vaginal birth	7.03 (6.90, 7.17)		4.87 (4.76, 4.98)	
Forceps	7.25 (6.98, 7.52)		5.70 (4.98, 6.43)	
Ventouse	7.18 (6.87, 7.49)		5.46 (4.82, 6.09)	
Caesarean section	7.14 (6.94, 7.33)	0.03	4.92 (4.72, 5.11)	0.04
*Number of positive adjectives describing staff in labour and birth*				
0-3	6.58 (6.38, 6.77)		4.64 (4.45, 4.82)	
4-5	7.06 (6.89, 7.23)		4.80 (4.64, 4.96)	
6+	7.48 (7.34, 7.63)	0.00	5.18 (5.03, 5.33)	0.00
*After birth, had skin-to-skin contact with baby*				
Yes	7.19 (7.08, 7.30)		4.92 (4.82, 5.02)	
No, not offered	6.85 (6.53, 7.16)		5.02 (4.63, 5.42)	
No, not well enough	6.76 (6.37, 7.15)		4.92 (4.44, 5.39)	
No, not wanted	5.87 (4.77, 6.97)	0.02	4.63 (3.98, 5.27)	0.72
*Staff communication in labour*				
Very/fairly well	7.14 (7.03, 7.24)		4.93 (4.83, 5.03)	
Not very/at all well	6.60 (6.23, 6.98)	0.30	4.63 (4.28, 4.98)	0.05
*Overall satisfaction with intrapartum care*				
Satisfied or very satisfied	7.16 (7.05, 7.26)		4.94 (4.84, 5.04)	
Neither satisfied nor dissatisfied	6.85 (6.40, 7.30)		4.53 (4.10, 4.96)	
Dissatisfied or very dissatisfied	6.54 (6.15, 6.94)	0.03	4.88 (4.50, 5.26)	0.08

*Adjusted for maternal age, ethnicity, IMD, age left full-time education, and father’s country of birth.

Other variables entered in analysis but not significant in either primiparous or multiparous women: staff spoke so woman could understand, were kind and respectful antenatally, woman stayed in hospital overnight antenatally; number of negative adjectives applied to staff in labour, treated as an individual, staff spoke so woman could understand, were kind and respectful in intrapartum period, induction of labour, duration of labour, able to move around in labour, type of caesarean section, held/breastfed soon after birth, had confidence in staff intrapartum, left alone in labour or soon after the birth when it was worrying, how woman felt in first few days after the birth.

After adjustment for sociodemographic factors, greater paternal engagement was positively associated with first contact with health professionals before 12 weeks gestation, earlier booking in multiparous women, and irrespective of parity, having a dating scan, number of antenatal checks and offer and attendance at antenatal classes. In multiparous women paternal engagement was also associated with increased number of antenatal health problems and worries about labour. Women with more engaged partners also used more positive adjectives to describe care during labour and birth and multiparous women with more engaged partners were more satisfied overall with their antenatal care. Other indicators of perception of antenatal care were unaffected.

Certain outcomes of care were also associated with paternal engagement in pregnancy and labour. Adjusted paternal engagement score was significantly higher in women who delivered by forceps than women who delivered normally. Primiparous women who had skin-to-skin contact with their babies soon after birth, those who were satisfied with intrapartum care overall, and multiparous women who felt that staff communicated well with them in labour also reported higher levels of paternal engagement compared with other women.

Paternal involvement after the birth was estimated using the postnatal score (Table 6). After adjustment, where postnatal involvement was highest, women reported significantly better overall health at 3 months (mean engagement score in multiparous women who were well 14.7 (95% confidence interval 14.5, 14.8) compared to those who were not well 13.6 (95% confidence interval 13.1, 14.1); primiparous women reported fewer health problems at one month and multiparous women reported fewer health problems at each time point. Postnatal problems were also considered in groups [24]. At one month postpartum, where paternal involvement was higher, multiparous women were less likely to report psychological symptoms (‘blues’, depression, anxiety), bodily symptoms (stress incontinence, backache, dyspareunia), and post-traumatic stress symptoms (flash-backs to labour or birth, sleep problems not related to the baby, and difficulties in concentrating). Primiparous women were more likely to have a postnatal check with their doctor if paternal involvement was higher.

**Table 6:** Paternal involvement after birth using postnatal paternal engagement score (95% confidence interval) and maternal problems by parity

	**Primiparous women**		**Multiparous women**	
	**Mean (95% CI)**	Adjusted* *p*	**Mean (95% CI)**	Adjusted* *p*
*How woman felt at time of survey*				
very/quite well	16.0 (15.9, 16.1)		14.7 (14.5, 14.8)	
not very well/ill	15.3 (14.8, 15.8)	0.00	13.6 (13.1, 14.1)	0.00
*Number of postnatal problems at 10 days*				
0,1	15.9 (15.7, 16.2)		14.6 (14.3, 14.8)	
2,3	16.1 (15.9, 16.3)		14.8 (14.5, 15.0)	
4+	15.9 (15.7, 16.1)	0.50	14.2 (13.8, 14.6)	0.04
*Number of postnatal problems at 1 month*				
0	16.1 (15.8, 16.3)		14.8 (14.6, 15.1)	
1	16.0 (15.8, 16.3)		14.6 (14.3, 15.0)	
2+	15.9 (15.7, 16.0)	0.05	14.2 (13.9, 14.4)	0.00
*Number of postnatal problems at 3 months*				
0	15.9 (15.8, 16.1)		14.7 (14.5, 14.9)	
1+	16.0 (15.8, 16.2)	0.99	14.3 (14.0, 14.5)	0.01
*Postnatal psychological problems at 1 month*				
yes	15.9 (15.7, 16.2)		14.1 (13.7, 14.4)	
no	16.0 (15.8, 16.1)	0.68	14.7 (14.5, 14.8)	0.00
*Postnatal PTSD type symptoms at 1 month*				
yes	15.9 (15.7, 16.1)		14.1 (13.7, 14.4)	
no	16.0 (15.8, 16.1)	0.21	14.7 (14.5, 14.8)	0.01
*Postnatal bodily problems at 1 month*				
yes	15.9 (15.7, 16.1)		14.3 (14.1, 14.6)	
no	16.0 (15.9, 16.2)	0.14	14.7 (14.5, 14.8)	0.02
*Postnatal check*				
Check carried out	16.0 (15.9, 16.1)		14.6 (14.4, 14.7)	
Check not carried out	15.5 (15.2, 15.9)	0.03	14.4 (14.0, 14.8)	0.39

PTSD Post Traumatic Stress Disorder.

*Adjusted for maternal age, ethnicity, IMD, age left full-time education, and father’s country of birth.

Other variables entered in analysis but not significant in either primiparous or multiparous women: visited at home by midwife, number of different midwives who visited, would have liked to see midwife more/less frequently at home, met midwife before, confidence in staff postnatally, choice about where to access care, groups of postnatal problems at 10 days and 3 months.

### Outcomes of care and paternity leave

Paternity leave was strongly associated with well-being at three months (Table 7). After adjustment for sociodemographic variables and mode of delivery, women were more likely to feel unwell at this time when their partner had either taken no time off at all or took more than two weeks off. Multiparous women whose partner took no paternity leave were significantly more likely to report depression at one month and three months than women whose partners took the standard two weeks leave.

**Table 7:** Outcomes of care by paternity leave and by parity (%), and binary logistic regression adjusting for sociodemographic variables and mode of delivery

	**Paternity leave**			
	**None**	**1-9 working days**	**2 weeks**	**More than**
	**(n = 1257)**	**(n = 702)**	**(n = 2041)**	**2 weeks (n = 589)**
**How woman felt physically at 3 months**				
Primiparous women**				
Very/quite well	23.6	13.7	49.5	13.2
Not well/ill	29.3	8.6	41.4	20.7
OR (95% CI) of being not well/ill	1 (ref)	0.50 (0.27, 0.90)*	0.67 (0.46, 0.97)*	1.31 (0.84, 2.04)
Multiparous women**				
Very/quite well	29.4	17.8	40.9	11.9
Not well/ill	39.5	14.1	35.1	11.2
OR (95% CI) of being not well/ill	1 (ref)	0.65 (0.43, 0.97)*	0.66 (0.49, 0.90)*	0.70 (0.45, 1.10)
**Depression at 1 month**				
*Primiparous women*				
Yes	27.7	7.8	47.0	17.5
No	23.9	13.6	48.9	13.6
OR (95% CI) of depression	1 (ref)	0.53 (0.28, .01)	0.87 (0.58, 1.29)	1.15 (0.70, 1.91)
*Multiparous women* **				
Yes	46.0	15.1	28.8	10.1
No	29.8	17.4	40.9	11.9
OR (95% CI) of depression	1 (ref)	0.61 (0.36, 1.02)	0.47 (0.31, 0.72)**	0.55 (0.30, 1.03)
**Depression at 3 months**				
*Primiparous women*				
Yes	26.8	7.0	49.3	16.9
No	24.1	13.4	48.7	13.8
OR (95% CI) of depression	1 (ref)	0.50 (0.18, 1.37)	0.97 (0.54, 1.74)	1.20 (0.57, 2.55)
*Multiparous women* **				
Yes	44.0	15.5	25.0	15.5
No	30.2	17.4	40.7	11.7
OR (95% CI) of depression	1 (ref)	0.61 (0.31, 1.17)	0.42 (0.24, 0.74)**	0.85 (0.43, 1.68)

* p < 0.05 **p ≤ 0.01.

### Father’s involvement and infant feeding

Further analyses were conducted to assess fathers’ influence on infant feeding: women were asked about infant feeding after the birth and at the time of the survey and, if they had breastfed, the duration of breastfeeding (Table 8). They were also asked about breastfeeding problems at 10 days, one month and three months. After adjustment, women whose partners were more engaged antenatally and in labour were more likely to breastfeed and to breastfeed for longer, significantly so for primiparous women. However, in women who were breastfeeding, breastfeeding problems at 10 days were more common in those whose partners were more engaged, significantly so in multiparous women. There were no differences at one and three months.

**Table 8:** Mean partner engagement score (95% confidence interval) and associated infant feeding patterns and breastfeeding problems, by parity

	**Primiparous women**		**Multiparous women**	
	**Mean (95% CI)**	Adjusted* *p*	**Mean (95% CI)**	Adjusted* *p*
Tried to breastfeed at least once	7.18 (7.08, 7.29)		4.95 (4.85, 5.06)	
Never tried to breastfeed	6.36 (6.05, 6.66)	0.00	4.75 (4.53, 4.97)	0.05
*Infant feeding in first few days*				
Breastfeeding only	7.25 (7.13, 7.38)		4.95 (4.84, 5.06)	
Breast and formula	6.98 (6.76, 7.20)		4.91 (4.63, 5.20)	
Formula only	6.61 (6.35, 6.86)	0.00	4.76 (4.56, 4.97)	0.10
*Infant feeding at 3 months*				
Formula	6.89 (6.75, 7.03)		4.84 (4.71, 4.97)	
Breastfeeding	7.42 (7.25, 7.58)		4.97 (4.81, 5.13)	
Both formula and breast	7.06 (6.82, 7.30)		4.95 (4.70, 5.21)	
Other	7.67 (5.85, 9.38)	0.00	5.00 (2.68, 7.32)	0.31
*Breastfeeding duration*				
didn’t breastfeed	6.44 (6.17, 6.70)		4.82 (4.61, 5.03)	
1-20 days	7.12 (6.88, 7.36)		4.82 (4.58, 5.06)	
21-98 days	6.99 (6.76, 7.22)		4.90 (4.67, 5.13)	
still breastfeeding	7.30 (7.16, 7.43)	0.00	4.96 (4.83, 5.10)	0.25
*Of those women breastfeeding: Breastfeeding problems at 10 days*				
Yes	7.22 (7.06, 7.38)		5.11 (4.91, 5.31)	
No	7.20 (7.03, 7.37)	0.86	4.85 (4.71, 4.99)	0.03

*Adjusted for maternal ethnicity, age, IMD. age on leaving full-time education, and father’s country of birth.

## Discussion

This study demonstrates that the association reported in earlier small scale studies holds up in this larger sample. Parity, age, ethnicity and deprivation were key factors affecting the father’s reaction to and degree of involvement with the pregnancy, as well as the likelihood of him being present for the labour and helping with the baby postnatally. In the postnatal period, partners of multiparous women were less likely to be involved in childcare than primiparous women. This is consistent with a study from the USA [25] which reported that first time fathers do more infant care. This is unsurprising and probably reflects the additional childcare needed in multiparous households. Women in lower socioeconomic groups were also more likely to have a partner that was actively involved in childcare, consistent with data reported in the Millenium Cohort Study (MCS) [26]. Conversely, partners of women from BME groups were significantly less likely to be involved postnatally. Data from the MCS suggest that this is particularly in Indian, Pakistani and Bangladeshi fathers who were significantly less likely to feed or change their baby’s nappy [26].

The woman’s reaction to the pregnancy was also strongly associated with partner support, consistent with earlier work in this area which stressed the importance of partner stability, dependability and support in the wantedness of the pregnancy [27]. After adjustment for sociodemographic factors, greater paternal engagement was associated with earlier first contact with a health professional during pregnancy, earlier timing of the booking appointment and the woman was more likely to have a dating scan. However, it should be borne in mind that recall, even of such salient events, may be compromised by time. Similarly, where paternal engagement was high women were more likely to be offered and to attend antenatal classes. This may be consequent upon early booking as demand for classes is high and provision inadequate in many parts of England [28].

With respect to perceptions of care, although women were asked throughout the survey whether they felt they were treated with respect and kindness and spoken to so that they could understand, treated as an individual, had confidence in the staff, and overall satisfaction, the only measure that was significantly associated with paternal engagement was the positive subscale of the adjective checklist used to describe the staff in labour and delivery.

Women who reported greater partner involvement antenatally also reported more antenatal problems and worries about labour. This may reflect the increased support that may be required in circumstances where a woman is anxious or unwell.

Outcomes of care varied only slightly by paternal engagement after adjustment for sociodemographic factors. Paternal engagement was significantly higher in women who had a forceps delivery compared to women who had a normal delivery. These women also had longer labours and were more likely to have been induced. It is possible that fathers who were more supportive and engaged were particularly distressed at seeing their partner in pain and exhausted, possibly prompting earlier intervention than men who were less engaged with the process. It is possible that they also felt more empowered to intervene on their partner’s behalf. This is supported by an Italian study, examining fathers’ experience during labour where their partner had an epidural. They found that, where an epidural was used, men felt significantly more helpful and involved and experienced less stress and anxiety [29]. Postnatal health, particularly in multiparous women, tended to be better in women whose partner was more involved at this stage. At three months women were significantly more likely to report feeling physically ‘very well or ‘quite well’ where their partner was more involved. Similarly, they had fewer postnatal problems and were more likely to have a postnatal check. These findings conflict with data from the MCS which found that fathers’ involvement in childcare was not associated with mothers’ higher satisfaction or reduced depression [26]. However, these analyses related to children at 9 months at which point circumstances may be different.

Paternity leave had a similar effect in that, after adjustment, women whose partners had taken no paternity leave were more likely to report feeling unwell or ill at three months, and multiparous women reported much higher rates of depression at one and three months (46% and 44% respectively of those whose partners had taken no leave). However, partners of women who were unwell or ill at this point were also more likely to have a partner who took more than two weeks off, perhaps reflecting the fact that these women would have needed more help. The difference by parity in father’s postnatal involvement may, as speculated by Hosking et al. [30], be due to men taking a greater role in care of older children. Paternity leave was highly correlated with the postnatal engagement score which is consistent with MCS data indicating that men who took more leave tended to be more involved in nappy changing, feeding and getting up in the night [31]. Breastfeeding in the first few days and at three months was also positively associated with paternal engagement, particularly in primiparous women. This is consistent with other research in this area which has found that where fathers are more involved breastfeeding rates are higher [32]. However, another small study suggested that bottle feeding allowed men the time to develop their relationship with their babies and was encouraged by some women [33]. In multiparous women who were breastfeeding at 10 days, breastfeeding problems were more common in women whose partners were more engaged. This could be interpreted as women who receive support and encouragement from their partner persevering with breastfeeding, even when it is more difficult or problems arise. Also, multiparous women may be more aware than primiparous women that 10 days is still early for lactation to be well-established.

Other outcomes that were examined but which were not significantly associated with paternal engagement after adjustment were: women’s physical health in the first few days and most of the individual postnatal health problems which included fatigue, backache and stress incontinence as well as anxiety and depression.

Limitations to this study include the response rate of 55.1%. There was under-reporting from women who were young, single, from a BME group and those living in deprived areas. Nevertheless, the sample size of 5332, 86% of whom provided information about their partner, is considerable. A further limitation of this type of survey is its cross-sectional nature. It is therefore not possible to ascribe causality to the associations reported. Although some men may have completed the section relating to fathers themselves, the majority were probably completed by women on their partner’s behalf. They may not accurately reflect fathers’ views or degree of involvement and may be completed such as to present their partner in a particular way. However, data reported in the MCS suggest that the correlation between mothers’ and fathers’ reported amount of domestic work undertaken by men was high, although fathers over-report and mothers under-report men’s involvement [26]. Similarly, the sociodemographic characteristics were predominantly those of the woman reflecting her pattern of parity, deprivation and ethnicity rather than her partner’s, although where both parents’ ages and country of birth were available, these were highly correlated. The partner engagement scores were based on what may be considered fairly crude markers for partner involvement and recall over the duration of pregnancy and the postnatal period which may have led to inaccuracy. Moreover, many men may be unable to be present at the various checks, scans and classes antenatally but still be very supportive of their partner. Similarly, in this study women who were not living with their baby’s father were excluded although it is acknowledged that non-resident fathers are generally still involved with their children to a certain extent [19].

This study therefore strengthens the literature on father involvement with pregnancy and childbirth which is mainly qualitative and has largely focused on white, middle class men or relatively small scale quantitative studies. The pronounced effect of parity is previously unreported except in a limited manner [25] and although not unexpected, this powerfully reflects the life changing nature of the birth of the first child and the transition to parenthood with a new role and identity [34,35], compared to the situation with the birth of second and subsequent children. The cultural differences associated with ethnicity, particularly in the delivery room are also consistent with the literature in this area [36-38] as are the variations by women’s age and degree of deprivation [7].

Implications for practice principally lie in the importance of health professionals recognising that women in some sociodemographic groups may be less supported by their partner and more reliant on staff. They may book later in pregnancy and, as a consequence, miss the window of opportunity for dating scans and other screening tests. They may also miss out on antenatal classes which may leave them even more unsupported in the postnatal period. Nevertheless, the vast majority of fathers are reportedly pleased or overjoyed in reaction to the pregnancy, and involved antenatally, in labour and postnatally. While women and their babies are the main focus of care it seems that there is room for yet greater engagement with fathers. Health professionals can have an active role in supporting and facilitating this by encouraging fathers to attend appointments and classes with their partners and, where possible, to direct some parts of antenatal education specifically at fathers.

## Conclusion

From the women’s point of view the majority of recent fathers are clearly actively engaged in pregnancy, childbirth and afterwards. The positive association with women themselves accessing maternity care during pregnancy and postnatally and with outcomes including breastfeeding, reinforce a position that values fathers and their important role in supporting women at this critical time in the lives of their partners and children.

Most fathers were very positive about their partner’s pregnancy; almost all were present for pregnancy ultrasound examinations and for labour. Three-quarters of fathers took paternity leave and, during the postnatal period, most fathers helped with infant care. Greater paternal engagement was positively associated with timing of first contact with health professionals, having a dating scan, number of antenatal checks, offer and attendance at antenatal classes, and breastfeeding. Paternity leave was also strongly associated with maternal well-being at three months postpartum.

This study demonstrates the considerable sociodemographic variation in partner support and engagement in the processes associated with pregnancy care, labour and birth and in the early days of parenting and caring for a young baby at home. It is important that health professionals recognise that women in some sociodemographic groups may be less supported by their partner and more reliant on staff providing maternity services and support and that this impacts on women’s needs and how and when they access care.

## Competing interests

The authors declare that they have no competing interests.

## Authors’ contributions

MR was responsible for designing the survey, oversaw the management and coordination and helped to draft the manuscript. JH conducted the analysis and drafted the manuscript. Both authors read and approved the final manuscript.

## Pre-publication history

The pre-publication history for this paper can be accessed here:

http://www.biomedcentral.com/1471-2393/13/70/prepub
